# A novel circRNA-miRNA-mRNA network identifies circ-YOD1 as a biomarker for coronary artery disease

**DOI:** 10.1038/s41598-019-54603-2

**Published:** 2019-12-04

**Authors:** Liu Miao, Rui-Xing Yin, Qing-Hui Zhang, Pei-Juan Liao, Yong Wang, Rong-Jun Nie, Hui Li

**Affiliations:** 1 Department of Cardiology, Institute of Cardiovascular Diseases, The First Affiliated Hospital, Guangxi Medical University, 6 Shuangyong Road, Nanning, 530021 Guangxi China; 2 Guangxi Key Laboratory Base of Precision Medicine in Cardio-cerebrovascular Disease Control and Prevention, 6 Shuangyong Road, Nanning, 530021 Guangxi China; 3 Guangxi Clinical Research Center for Cardio-cerebrovascular Diseases, 6 Shuangyong Road, Nanning, 530021 Guangxi China; 4 Clinical Laboratory of the Affiliated Cancer Hospital, Guangxi Medical University, 71 Hedi Road, Nanning, 530021 Guangxi China

**Keywords:** Cardiovascular genetics, Genetic markers, Diagnostic markers

## Abstract

Circular RNAs (circRNAs) are involved in many physiological functions. Whether circulating circRNAs serve as markers for coronary artery disease (CAD) is unknown. Seven CAD-related microarray datasets were downloaded from the Gene Expression Omnibus (GEO) database and were analyzed using clustering and functional enrichment to identify hub mRNAs and miRNAs. StarBase V3.0 and circinteractome databases were used to predict interactions between circRNAs and miRNAs whereas miRwalk and DIANA TOOLS were used to predict interactions between miRNAs and mRNAs. Altogether, this helped establish a circRNA-miRNA-mRNA triple network for diagnosis of CAD. Five non-coding RNAs (ncRNAs) were identified in our study population with the use of quantitative real-time PCR (RT-PCR). The prognostic values of circYOD1, hsa-miR-21-3p and hsa-miR-296-3p were evaluated using a receiver operating characteristic (ROC) curve. A CAD circRNA-miRNA-mRNA network was established from our analyses containing one circRNA, four miRNAs and thirteen mRNAs. After performing RT-PCR validation between CAD and non-CAD samples, only three ncRNAs of five ncRNAs showed significance for further analysis. The area under ROC curve (AUC) of circ-YOD1 was 0.824, the AUC of hsa-miR-21-3p was 0.731 and hsa-miR-296-3p was 0.776. The pairwise comparison results showed that circ-YOD1 had statistical significance (*P*_YOD1-21_ < 0.01 and *P*_YOD1-296_ < 0.05). The results of functional enrichment analysis of interacting genes and microRNAs showed that the shared circ-YOD1 may act as a new biomarker for CAD. Our investigation of the triple regulatory networks of circRNA-miRNA-mRNA in CAD revealed circ-YOD1 as a potential biomarker for CAD.

## Introduction

Coronary artery disease (CAD), a complex and multifactorial disorder, remains one of the most common causes of death and results in a heavy economic and social burden worldwide^1^. Many environmental and genetic factors contribute to CAD, including age, smoking behavior, hypertension, dyslipidemia, obesity, diabetes and family history^2–5^. If there was a biomarker that can be measured in peripheral blood, it can be used for early detection of those at high-risk for CAD, which will help prevent and treat the disease^6^. However, at this stage, there is a limited number of biomarkers used in clinical practice to predict the incidence of CAD^7^. At present, the gold standard for the diagnosis of CAD is still coronary angiography. However, peripheral blood biochemical tests are only valuable for assessing the risk factors of CAD. And these examinations usually show significant changes in severe complications, such as acute coronary syndrome. Hysteresis, non-specificity and other factors limit the application of traditional examination items in clinical practice.

Most noncoding RNAs (ncRNAs) have been involved in the regulation of many physiological and pathological processes^8^. Circle RNAs (circRNAs) result when an exon of a gene is spliced backwards from the 5′ to 3′ segment^9^. Previous studies have shown that circRNAs may modify parental gene expression through their function as microRNA (miRNA) sponges^10^. This way, circRNAs play an important role in the pathogenesis and progression of CAD, which deserves in-depth investigation^11^.

**Figure 1:**
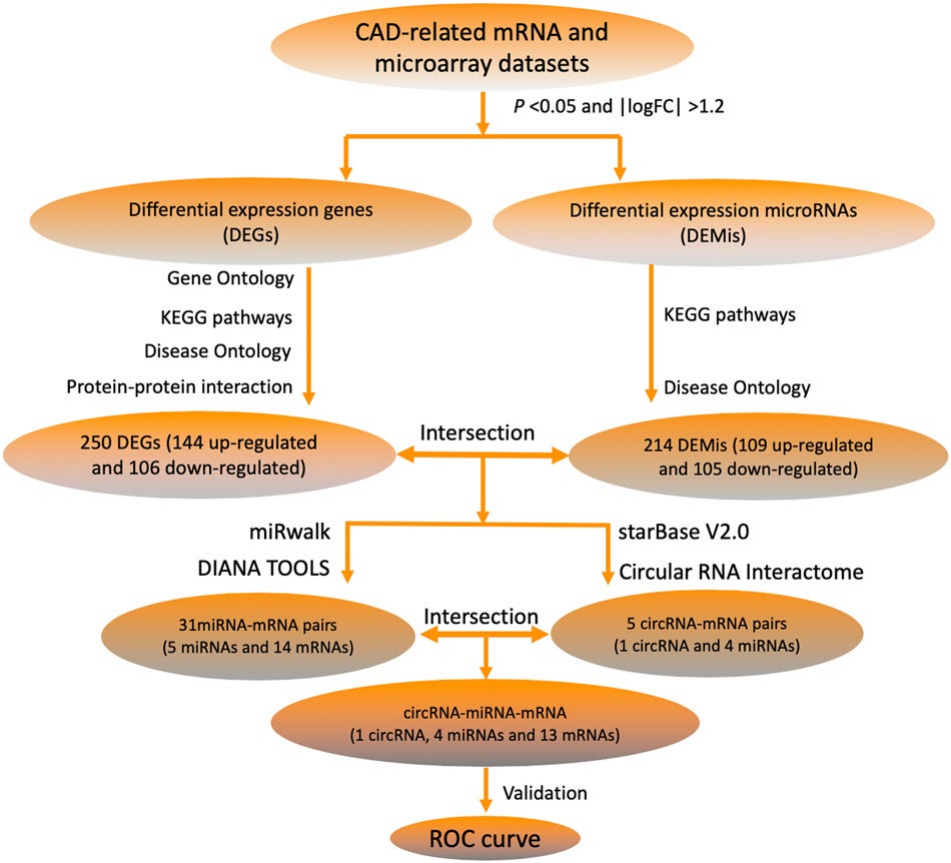
A flowchart of ceRNA network construction.

Recent studies have shown that certain ncRNAs affect physiological and pathological processes of CAD^12^. Since ncRNAs are stable in peripheral blood and other body fluids, they serve as strong potential predictive markers for CAD. Our study is based on the hypothesis that some ncRNAs can be used as a biomarker for CAD. In this study, a CAD triple network of circRNA-miRNA-mRNA was established though mapping differentially expressed items using data downloaded from the Gene Expression Omnibus (GEO) repository^13^ and by identifying which ncRNAs are specific biomarkers for CAD. The flowchart is shown in Fig. 1.

## Results

### Microarray preprocessing

After quality control and elimination of incorrect expression data, we identified a total of 250 DEGs, including 144 that were upregulated and 106 downregulated DEGs in CAD samples as compared to control samples. Both the heatmap and volcano plot are shown in Supplementary Fig. 1. A total of 214 DEMis, including 109 that were upregulated and 105 downregulated DEMis, are shown in the heatmap and volcano plot in Supplementary Fig. 2. Hsa-miR-361-5p was found in GSE53211, and hsa-miR-21-3p, hsa-miR-296-3p and hsa-miR-375 were found in GSE61741. In addition, GSE34198 consisted of the DEGs *CHRM1*, *FBXL18*, *FGF2*, *GPR17* and *IL1A*; GSE60993 consisted of *MMP9*, *FCGR3B* and *GZMK;* and GSE61144 consisted of *BCL6*, *GZMA*, *MZMK*, *MMP9*, *EOMES* and *IL2RB*.

### Hub items identification

**Figure 2:**
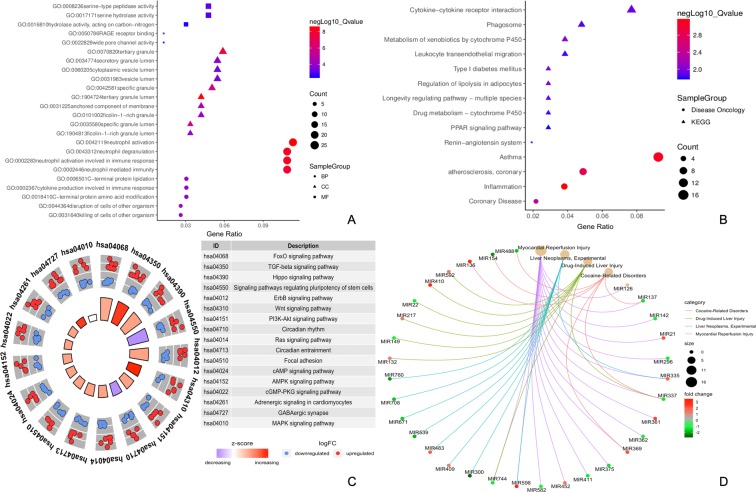
Functional annotation for DEGs and DEMis. (**A**) Gene Ontology for DEGs; (**B**) KEGG pathway and Disease Ontology for DEGs; (**C**) KEGG pathway for DEMis. The height of the bar in the inner ring provides the significance of the term and color and corresponds to the Z-score. The scatter plots in outer ring indicate the expression levels (logFC) for the genes in each term. (**D**) Disease Ontology for DEMis.

**Figure 3:**
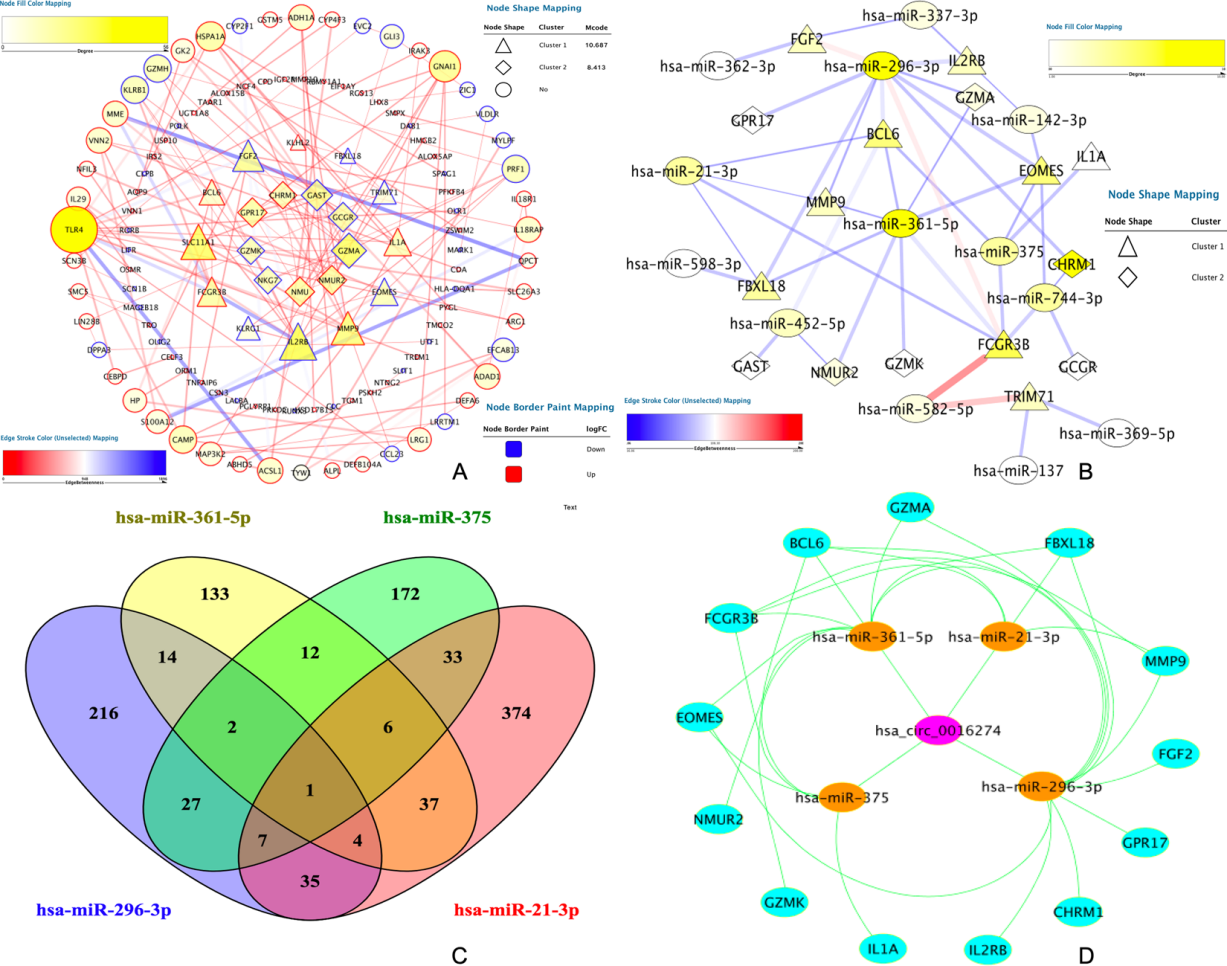
Protein-protein interaction analysis and construction of the circRNA-miRNA-mRNA network. (**A**) Protein-protein interaction analysis of differentially expressed genes. Edge stands for the interaction between two genes. The more edge, the bluer the node. The importance of protein nodes in the network is described by degree where white denotes low and yellow denotes high. The molecular complex detection method (MCODE), as the significant modules, picked up from the PPI network by score of >6.0. Significant modules were identified from the PPI network using the molecular complex detection method with a score of >6.0. Unique shapes represented different clusters where triangles indicate cluster 1 and diamonds signifying cluster 2. The border color represents the fold change for DEGs where upregulated DEGs are marked red and downregulated DEGs marked blue. (**B**) PPI network for DEGs and DEMis. Edge stands for the interaction between two genes. The more edge, the redder the node. The importance of protein nodes in the network is described by degree where white denotes low and yellow indicates high. Unique shapes represent different clusters, and triangles indicates cluster 1, with diamonds signifying cluster 2. (**C**) Venn diagram for the overlap of numbers of the predicted circRNAs. (**D**) CircRNA-miRNA-hub gene network. Hsa_circ_0016274 as an item of circ-YOD1.

We analyzed all 250 genes using the R clusterProfiler package to elucidate their roles in GO, DO and KEGG. The result is shown in Fig. 2A,B. All DEGs are presented in Supplementary Table 1. The same method was performed to analyze miRNAs (Supplementary Table 2). After KEGG and DO functional analyses (Fig. 2C,D), 16 hub miRNAs were identified for further studies. When using the STRING database for analysis, 121 nodes and 160 protein pairs with a combined weight score >0.25 were screened in the DEGs (Fig. 3A). When they were analyzed in the submodule, only two modules with a score >6 were detected by MCODE. In these two clusters, the MCODE score for cluster 1 was 10.687, including 12 DEGs; however, the MCODE score for cluster 2 was 8.413, consisting of 9 DEGs.

### Construction of the ceRNA regulatory network

**Figure 4:**
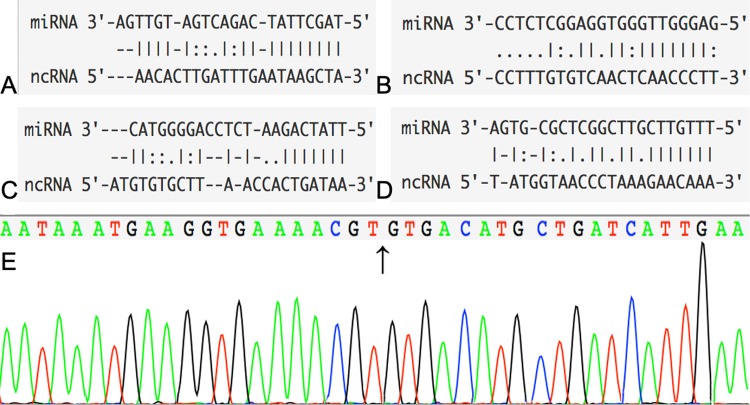
Interactions of miRNAs and circRNAs and verification of the RT-qPCR product of circ-YOD1. CircRNA-miRNA interactions identified by both the starBase and Circinteractome databases. (**A**) hsa-miR-21-3p; (**B**) has-miR-296-3p; (**C**) has-miR-361-5p; (**D**) has-miR-375. (**E**) Head-to-tail splicing in the RT-qPCR product of circ-YOD1.

MiRwalk and DIANA TOOLS databases were used to predict the interaction between microRNAs and mRNAs. If the predicted genes in the database were not DEGs, then they were removed. Using this technique, we identified 44 miRNA-mRNA interaction pairs (Fig. 3B). Next, we predicted circRNAs that could bind with miRNAs to design the circRNA-miRNA regulatory network using the starBase (V3.0) and circinteractome databases. After this, only one circRNA could intersect with four miRNAs (Fig. 3C). Previous work has found that circRNAs play critical roles as “decoys” to sponge miRNAs. Figure 4A–D shows binding sites for circRNAs and miRNAs. Finally, a circRNA-miRNA-mRNA triple regulation network was established by the integrated two-interaction network and visualized by Cytoscape (Version 3.61). MRNAs, miRNAs and circRNAs were represented as nodes in the same network. The edges represented interactions between items^14^ in the network (Fig. 3D). Finally, we found that circRNA-YOD1 and four miRNAs (hsa-miR-21-3p, hsa-miR-296-3p, has-miR-361-5p and hsa-miR-375) were hub items in this triple regulatory network. The head-to-tail splicing in the RT-qPCR product of circ-YOD1 was confirmed by Sanger sequencing and is shown in Fig. 4E.

### Validation of expression profiles

**Figure 5:**
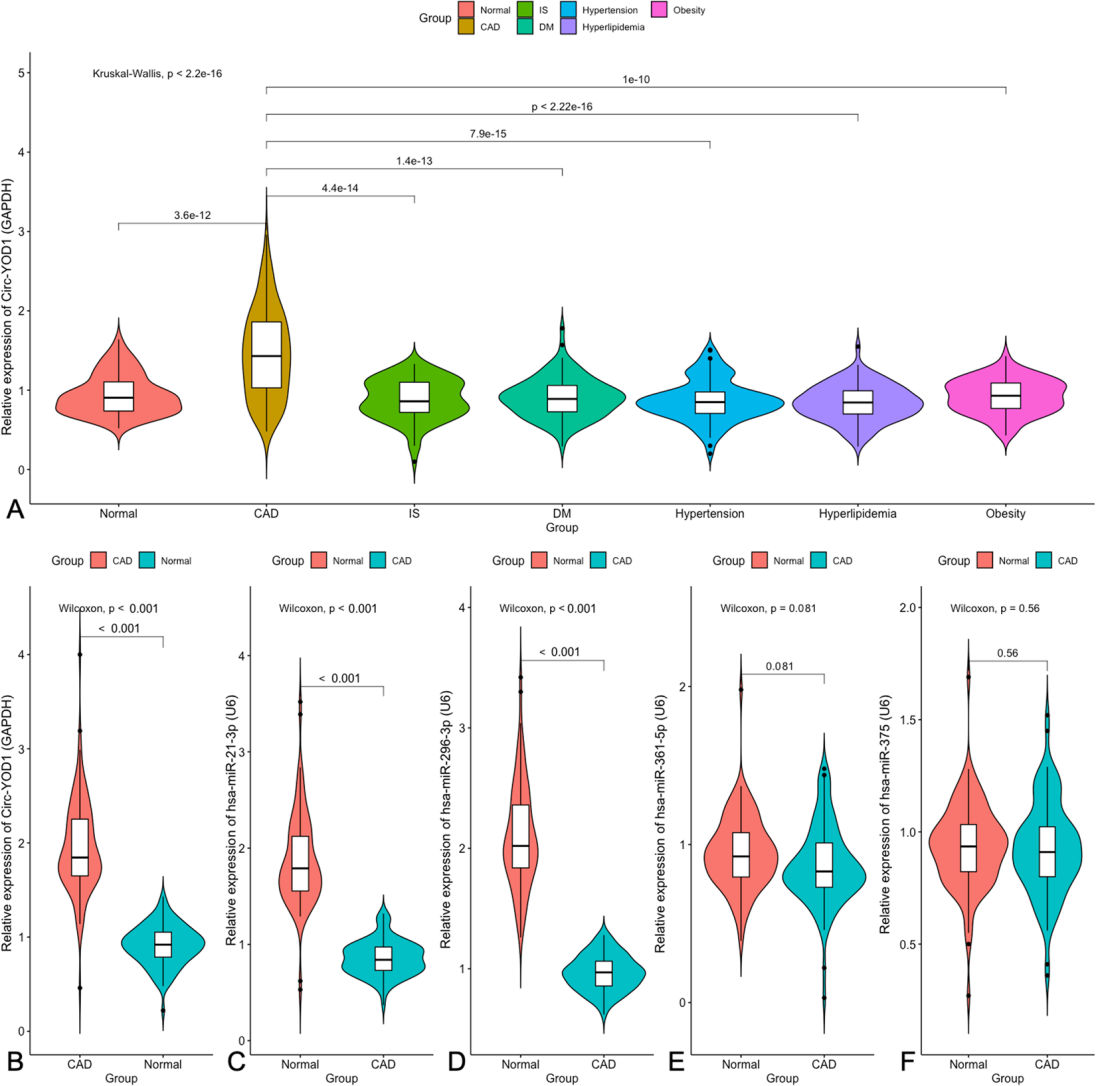
Confirmed expression levels of ncRNAs potentially involved in CAD. (**A**) The relative expression of circ-YOD1 among diseases associated with CAD. (**B**–**F**) The relative expression of ncRNAs in CAD samples. Abbreviations were as follows: *CAD*, Coronary artery disease; *IS*, Ischemic stroke; *DM*, Diabetes mellitus.

After a comprehensive analysis, the predicted circRNA was validated in our samples, including 316 controls and 1842 patients who suffered from CAD. Demographic results are summarized in Table 1. After validation, we found that the related expression of circRNA-YOD1 was significantly increased in CAD patients compared to controls (Fig. 5A). Subsequently, 1 circRNA and 4 miRNAs were selected for validation in another CAD sample, including 323 patients and 342 controls. The two groups were matched for gender and age, and the details are shown in Table 2. Compared to controls, we found that the related expression of circ-YOD1 was significantly increased in CAD patients, but hsa-miR-21-3p and hsa-miR-296-3p were reduced (Fig. 5B–F). The two microRNAs are predicted in starBase (V3.0) with interactions supported by Ago CLIP-seq data.

**Table 1:** Comparison of demographic, lifestyle characteristics and serum lipid levels between the normal and case groups.

Parameter	Control	CAD	IS	DM	Hypertension	Dyslipidemia	Obesity
Number	316	322	308	296	314	298	304
Male/female	95/221	90/232	96/212	88/208	100/214	88/210	95/199
Age (years)^1^	55.31 ± 8.42	54.17 ± 10.03	56.56 ± 8.22	55.93 ± 9.94	54.96 ± 10.22	56.13 ± 8.23	54.67 ± 9.65
Height (cm)	156.25 ± 6.81	158.53 ± 7.13	157.61 ± 7.43	156.49 ± 6.83	161.19 ± 9.23	157.44 ± 7.16	156.78 ± 6.43
Weight (kg)	52.86 ± 7.84	56.74 ± 10.84^a^	57.52 ± 9.69^a^	57.88 ± 8.81^a^	56.12 ± 9.81^a^	56.79 ± 10.42^a^	63.81 ± 11.22^b^
Body mass index (kg/m^2^)	29.59 ± 5.23	30.31 ± 6.54	30.66 ± 7.23	29.79 ± 5.78	30.27 ± 6.09	30.13 ± 5.52	32.31 ± 7.98^b^
Waist circumference (cm)	73.43 ± 6.61	75.45 ± 9.69	77.22 ± 8.87	76.41 ± 8.83	77.15 ± 9.27	77.65 ± 7.78	87.45 ± 9.87^c^
Smoking status [*n* (%)]	82 (26.0)	112 (34.8)^a^	111 (36.3)^a^	105 (35.4) ^a^	113 (36.1)^a^	86 (30.4)^a^	95 (31.2)^a^
Alcohol consumption [*n* (%)]	75 (23.9)	82 (25.5)	76 (24.6)	86 (28.8) ^a^	76 (24.3)	75 (25.2)	79 (26.1)^a^
SBP (mmHg)	128.24 ± 18.18	129.47 ± 22.16^a^	130.05 ± 22.96^a^	128.13 ± 20.14	147.55 ± 26.16^c^	128.49 ± 20.22	129.11 ± 20.55
DBP (mmHg)	81.54 ± 10.16	82.49 ± 13.15	85.54 ± 14.21^a^	81.56 ± 13.23	93.54 ± 15.15^c^	82.08 ± 12.45	82.54 ± 13.23
PP (mmHg)	49.64 ± 14.13	50.42 ± 14.59	50.66 ± 15.14	49.83 ± 15.19	55.61 ± 17.55^c^	50.13 ± 13.53	51.11 ± 14.44
Glucose (mmol/L)	5.92 ± 1.86	6.03 ± 2.07	6.09 ± 2.05	8.45 ± 2.25^c^	5.99 ± 2.32	6.12 ± 1.95	6.09 ± 2.33
TC (mmol/L)	4.91 ± 1.13	5.24 ± 1.07^a^	5.33 ± 1.22^a^	4.99 ± 1.17	5.01 ± 1.02	4.99 ± 1.03	4.97 ± 1.17
TG (mmol/L)^2^	1.49 (0.51)	1.53 (1.22)	1.52 (1.21)	1.44 (1.32)	1.51 (1.26)	1.88 (2.23)^b^	1.72 (1.76)^a^
HDL-C (mmol/L)	1.52 ± 0.44	1.32 ± 0.26^a^	1.35 ± 0.34^a^	1.44 ± 0.28^a^	1.48 ± 0.32	1.06 ± 0.34^b^	1.46 ± 0.29
LDL-C (mmol/L)	2.84 ± 0.84	2.99 ± 0.79^a^	2.98 ± 0.77^a^	2.88 ± 0.76	2.92 ± 0.84	2.95 ± 0.92	2.86 ± 0.75
ApoA1 (g/L)	1.23 ± 0.25	1.18 ± 0.27	1.19 ± 0.25	1.17 ± 0.26	1.19 ± 0.25	1.14 ± 0.33^a^	1.20 ± 0.29
ApoB (g/L)	0.83 ± 0.19	0.89 ± 0.32	0.88 ± 0.31	0.91 ± 0.31	0.86 ± 0.28	0.87 ± 0.31	0.89 ± 0.29
ApoA1/ApoB	1.67 ± 0.50	1.66 ± 0.57	1.64 ± 0.58	1.65 ± 0.61	1.67 ± 0.54	1.66 ± 0.49	1.65 ± 0.43

*CAD*, coronary artery disease; *IS*, ischemic stroke; *DM*, diabetes mellitus; *SBP*, systolic blood pressure; *DBP*, diastolic blood pressure; *PP*, pulse pressure; *TC*, total cholesterol; *TG*, triglyceride; *HDL-C*, high-density lipoprotein cholesterol; *LDL-C*, low-density lipoprotein cholesterol; *Apo*, Apolipoprotein. ^1^Mean ± SD determined by *t*-test. ^2^Because of not normally distributed, the value of triglyceride was presented as median (interquartile range), the difference between the two groups was determined by the Wilcoxon-Mann-Whitney test. The *P* value was defined as the comparison of case and control groups. ^a^*P* < 0.05; ^b^*P* < 0.01; ^c^*P* < 0.001.

**Table 2:** Comparison of demographic, lifestyle characteristics and serum lipid levels between the normal and CAD groups.

Parameter	Control	CAD	*test-statistic*	*P*
Number	323	342		
Male/female	98/225	108/234	0.675	0.409
Age (years)^1^	55.32 ± 10.42	55.88 ± 10.13	0.954	0.393
Height (cm)	155.23 ± 6.92	155.58 ± 7.12	1.594	0.222
Weight (kg)	52.85 ± 7.64	60.54 ± 10.82	20.439	1.67E-005
Body mass index (kg/m^2^)	29.49 ± 3.13	32.30 ± 6.24	28.214	2.21E-008
Waist circumference (cm)	73.43 ± 6.61	87.45 ± 9.87	23.122	3.34E-005
Smoking status [*n* (%)]	84 (26.0)	119 (34.8)	7.690	0.005
Alcohol consumption [*n* (%)]	77 (23.9)	87 (25.5)	0.309	0.578
Systolic blood pressure (mmHg)	129.23 ± 18.18	129.47 ± 21.16	0.413	0.432
Diastolic blood pressure (mmHg)	80.54 ± 10.16	82.49 ± 13.35	0.717	0.291
Pulse pressure (mmHg)	49.64 ± 14.13	50.42 ± 14.59	1.492	0.233
Glucose (mmol/L)	5.91 ± 1.76	7.64 ± 2.73	17.867	5.02E-005
Total cholesterol (mmol/L)	4.93 ± 1.13	5.34 ± 1.16	7.131	0.016
Triglyceride (mmol/L)^2^	1.49 (0.51)	1.53 (1.22)	2.137	0.187
HDL-C (mmol/L)	1.52 ± 0.44	1.06 ± 0.26	8.673	0.013
LDL-C (mmol/L)	2.84 ± 0.84	2.88 ± 0.79	9.497	0.007
ApoA1 (g/L)	1.23 ± 0.25	1.17 ± 0.27	0.384	0.518
ApoB (g/L)	0.83 ± 0.19	0.89 ± 0.32	1.542	0.193
ApoA1/ApoB	1.67 ± 0.50	1.66 ± 0.57	0.095	0.758

*HDL-C*, high-density lipoprotein cholesterol; *LDL-C*, low-density lipoprotein cholesterol; *Apo*, Apolipoprotein. ^1^Mean ± SD determined by *t*-test. ^2^Because of not normally distributed, the value of triglyceride was presented as median (interquartile range), the difference between the two groups was determined by the Wilcoxon-Mann-Whitney test.

### Functional significance of validated circRNAs

**Figure 6:**
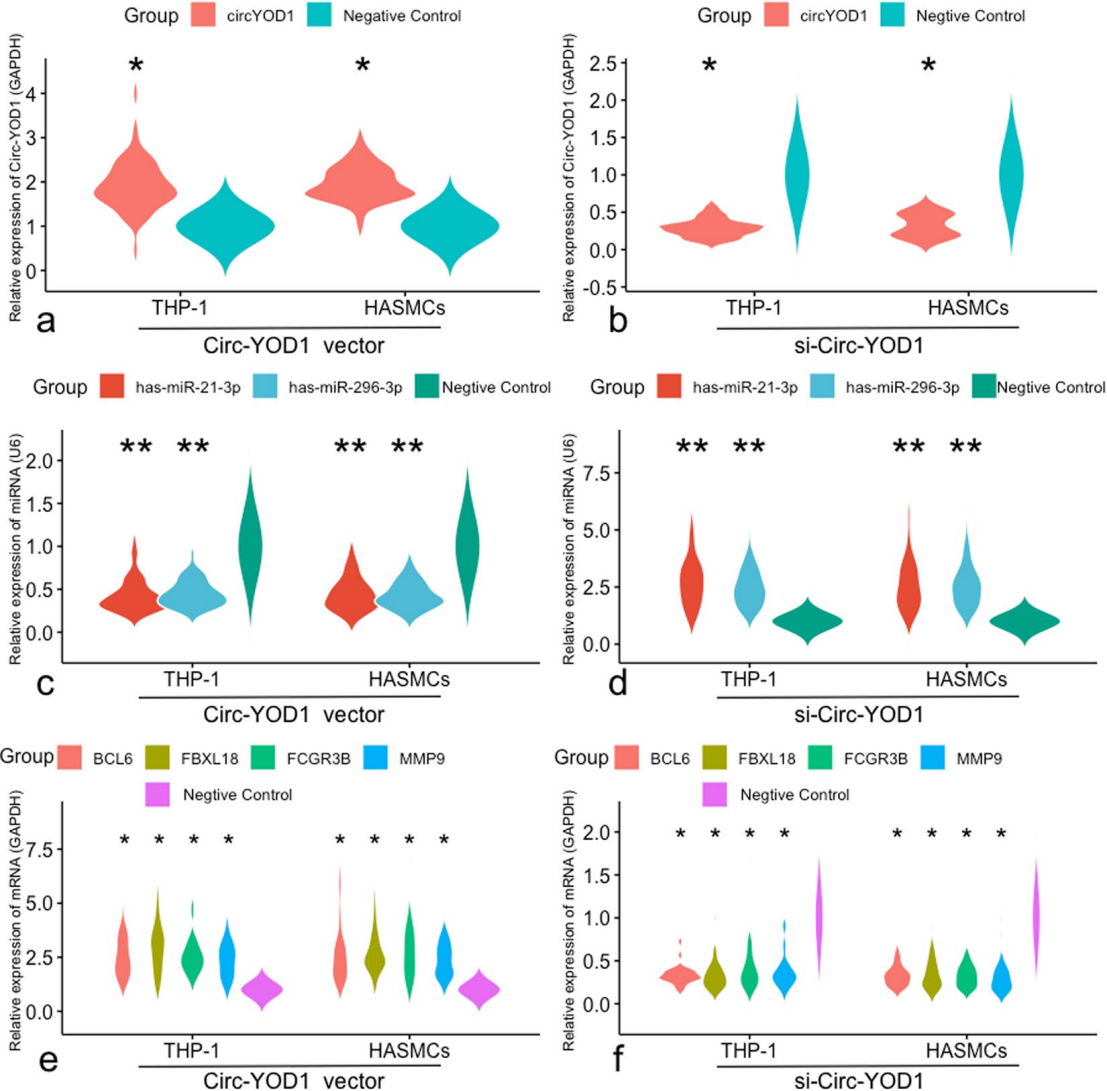
The regulatory role of the circRNA-miRNA-hub gene axes in THP-1 and HASMC cells. (**a**,**b**) Expression levels of circ-YOD1 were confirmed relative to control by qRT-PCR analysis after transfection. (**c**,**d**) Expression levels of miR-21-3p and miR-296-3p were regulated by circ-YOD1 compared to negative control. (**e**,**f**) Expression of target genes mediated by the circ-YOD1-miR-21-3p/miR-296-3p axis. *P < 0.01, **P < 0.001.

Prior studies have demonstrated that circRNAs can sponge multiple miRNAs in CAD. Therefore, we hypothesized that circ-YOD1 could play a negative regulatory role on miR-21-3p/miR-296-3p expression in CAD. After transfection with siRNA or the circ-YOD1 overexpression vector, circ-YOD1 expression was identified using qRT-PCR in THP-1 cells and HASMCs (Fig. 6A,B). Then, the expression levels of miR-21-3p and miR-296-3p were downregulated significantly in the overexpression circ-YOD1 group compared to the negative control. Accordingly, downregulation of circ-YOD1 expression could increase the expression of miR-21-3p and miR-296-3p as analyzed after transfecting THP-1 cells and HASMCs with siRNAs against circ-YOD1 (Fig. 6C,D). Finally, we annotated the target genes of the circ-YOD1-miR-21-3p/miR-296-3p axes. The expression levels of *BCL6*, *FBXL18*, *MMP9* and *FCGR3B* were significantly upregulated in the overexpression circ-YOD1 group compared with the negative control. Accordingly, downregulation of circ-YOD1 expression could reduce the expression of *BCL6*, *FBXL18*, *MMP9* and *FCGR3B* as shown by transfecting THP-1 cells and HASMCs with siRNAs (Fig. 6E,F).

### Plasma levels of ncRNAs sensitive to CAD

**Figure 7:**
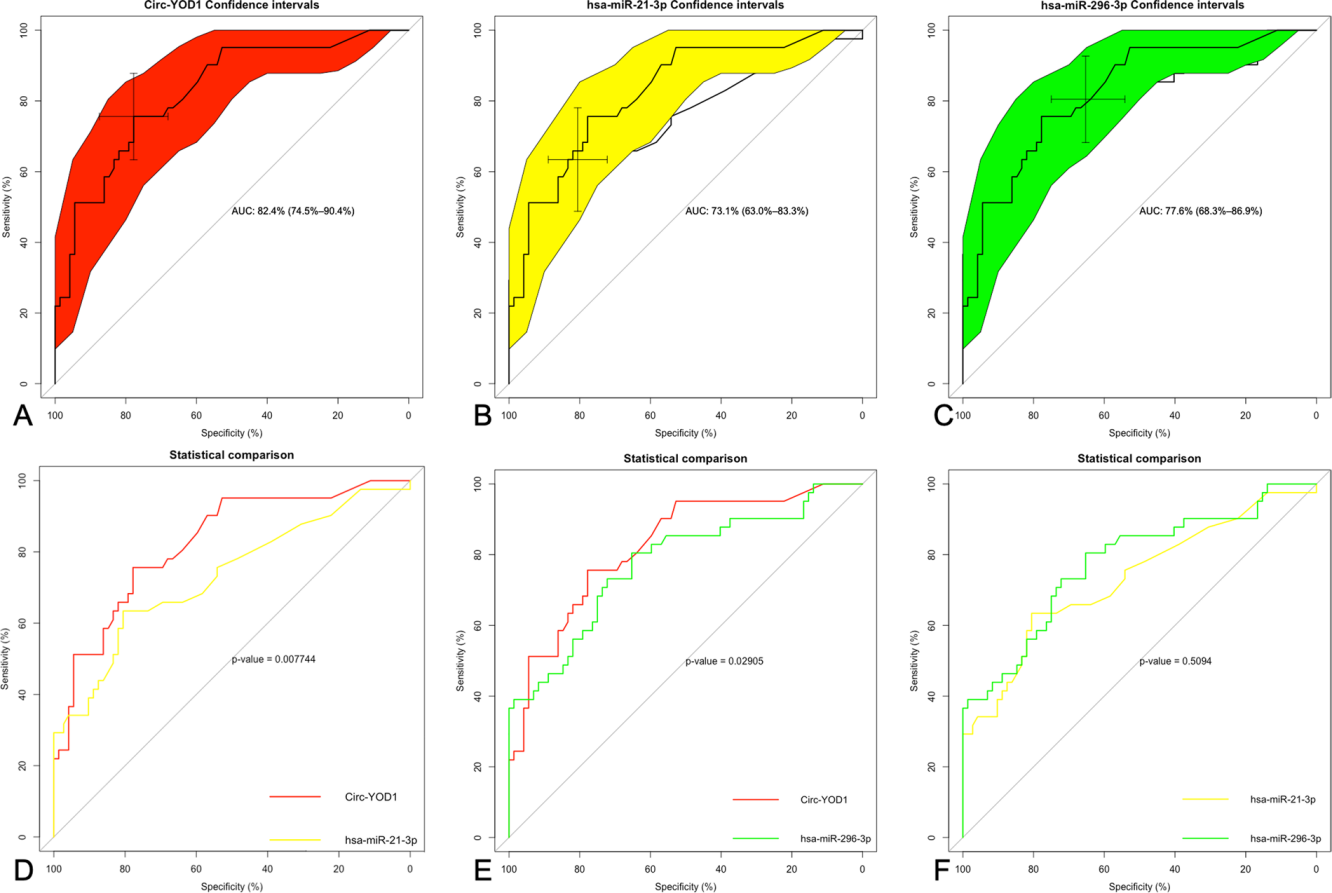
ROC curve analyses of 3 ncRNAs that can serve as biomarker diagnoses of CAD. (**A**) ROC curve analysis of circ-YOD1, (**B**) hsa-21-3p, (**C**) hsa-269-3p as well as (**D**–**F**) pairwise *P*-value comparison.

Based on our observations, we further explored the 3 ncRNAs as markers for CAD. The result of ROC curve analysis showed that the 95% confidence interval (CI) of circ-YOD1 was 0.745–0.904, and the AUC was 0.824. The AUC and 95% CI of hsa-miR-21-3p were 0.731 and 0.630–0.833, and those of hsa-miR-296-3p were 0.776 and 0.68.3–0.869; respectively (Fig. 7A–C). The results of pairwise comparison showed a statistically significant difference for circ-YOD1 (*P*_YOD1-21_ < 0.01; *P*_YOD1-296_ < 0.05, Fig. 7D–F).

## Discussion

Identifying the biological mediators of a disease can not only improve our understanding of its pathogenesis but also suggest new strategies to prevent and/or treat a disease, such as CAD. With remarkable improvements in social living standards and technological development, numerous biomarkers associated with CAD have been identified for the prevention of CAD^15^. However, this condition is not easy to identify in its primary stage by regular examinations, such as cardiac ultrasound and electrocardiography. Treatment identification and detection of CAD in its early stage with a sensitive biomarker are important. Recent large-scale studies have suggested that circRNAs play an indispensable role in the pathogenesis and progression of CAD^16,17^. Thus, we performed an integrated bioinformatic analysis and speculated that circ-YOD1 may serve as a diagnostic or prognostic biomarker of CAD and have potential therapeutic value.

Previous studies have confirmed that circRNAs can act as a molecular sponge of microRNA, where they competently bind, alter gene expression and interfere with selective gene splicing and transcription^18^. The roles in cardiovascular diseases are as follows: (1) protection of the heart from pathological hypertrophy and heart failure^19^; (2) induction of myocardial infarction^20^; (3) promotion of cardiac senescence^21^; and (4) correlation with atherosclerosis risk^22^. Recent studies have shown that YOD1 may affect systolic blood pressure and diastolic blood pressure^23^ and can disrupt the interaction between cardiomyocytes through miR-21^24^. In addition, YOD1 can bind to the C-terminal TRAF homology domain of TRAF6, which also serves as the interaction surface for the adaptor p62/Sequestosome-1required for IL-1 signaling to NF-κB. Interestingly, the NF-κB pathway is closely related to CAD^25,26^. Thus, Circ-YOD1 may regulate parental gene YOD1 expression that leads to CAD.

MiRNAs may also play crucial roles in the pathogenesis of CAD, and two of the miRNAs identified in our study have also been related to CAD based on prior work^27,28^. In addition, these miRNAs can act on CAD-related genes and affect gene expression, just as miR-21 and *MMP9*^29^. Although miRNAs were a small part of the non-coding transcriptome, the entanglement of circRNAs may contain undetermined molecular classes, so as a diagnostic tool, circRNAs have relatively advantageous properties over miRNAs. According to our results, circRNAs can be used as a potential biomarker to predict the diagnosis of CAD. In this study, we measured and compared the relative expression of circRNA in healthy people and patients with CAD. By comparison, we found that the relative expression of circYOD1 in patients with CAD increased significantly, and the specificity of diagnosis of CAD reached 82.4%.

Recently, the circRNA-miRNA-mRNA axis was discovered to regulate the development of CAD. For instance, the *TRPM3* could regulate the proliferation and contractility of vascular smooth muscle cells in coordination with cholesterol, perhaps potentially involved in therapeutic vascular modulation^30^ and as a target regulated by hsa-miR-130a-3p. In addition, 9 circRNAs could promote *TRPM3* expression by inhibiting hsa-miR-130a-3p in CAD patients through the circRNA-miRNA-mRNA axis^31^. These results further indicate that circRNAs can be used as a predictive marker for disease diagnosis, providing new targets and therapeutic ideas for the prevention and treatment of CAD in the future, and providing a variety of options for the prevention and treatment of disease in the future. In our present study, we found that several DEGs were associated with CAD, such as *MMP9* and *BCL6*^29,32^, and were targets for hsa-miR-21-3p and hsa-miR-296-3p. Two of these miRNAs were related to CAD^27,29^ and can be regulated by circ-YOD1. CircRNAs produced by genomic alternative splicing represent a special class of noncoding RNA molecules that are abundant in the cytoplasm of eukaryotic cells, have characteristic tissues, time of action and disease specificity and are highly conserved among different species^33^. Due to the biological characteristics of the covalently closed loop of circRNA, it is resistant to RNase degradation, and its properties are particularly stable compared to linear RNA. At the same time, noncoding RNA has relatively stable expression in plasma or in serum. Therefore, this circular RNA may become a potential biomarker^34,35^. Our *in vitro* cell experiments also verified this ceRNA relationship. These results indicate that circ-YOD1 may be a good predictive candidate biomarker for the diagnosis of CAD.

We also have to acknowledge that there are some downfalls to these analyses. First, all study subjects came from the same hospital and this study is a cross-sectional observational study. Whether the above conclusions can be validated by different ethnic groups in different regions is still unknown. Therefore, a prospective experiment is needed to verify the validity of the diagnostic efficacy of circYOD1 for CAD. Second, without a sufficient luciferase assay, we only identified circ-YOD1 as a biomarker for CAD. The specific mechanism of the circRNA-miRNA-mRNA axis for regulating the pathogenesis of CAD has not been fully validated both *in vivo* and *in vitro*. Third, due to sample limitations, we were unable to further verify the results in serum. All of the above gagners support to conduct further experiments to ensure the reliability of the conclusions.

In conclusion, we established a triple regulatory network of circRNA-microRNA-mRNA, detected the molecular mechanism of CAD, and finally determined that circYOD1 could be used as a good candidate marker to predict the onset of CAD.

## Materials and Methods

### Reannotation of gene expression profile probes

Seven gene-expression datasets were selected for further bioinformatic analysis, all downloaded from the Gene Expression Omnibus database (https://www.ncbi.nlm.nih.gov/geo/), including GSE34918^36^, GSE62646^37^, GSE60993 and GSE61144^38^ that were used to study mRNAs and GSE24548, GSE53211 and GSE53675^39^ used to study miRNAs. The primary species of these datasets were associated with CAD. All analyzed samples in the microarray were taken from peripheral blood. The details for each microarray are shown in Supplementary Table 3. First, the Affy package in R^40^ was used to convert the CEL file matrix, which is unified in the form of expression value. Next, the Robust Multi-array Average (RMA) method was performed to normalize the expression matrix. Finally, the Bioconductor package in R was employed to convert the probe information into a gene symbol^41^. When multiple probes corresponded to only one gene, the average between the probes were used for the final expression.

### Differentially expressed items and functional enrichment analysis

Differentially expressed mRNAs (DEGs) and miRNAs (DEMis) were selected by using the limma package^42^ in R. The threshold points were set to *P* < 0.05 and |log 2-fold-change| ≥ 1.2 for both DEGs and DEMis. The disease ontology (DO), gene ontology (GO) and Kyoto Encyclopedia of Genes and Genomes (KEGG) pathway analyses were used as well as the clusterProfiler, DOSE and the Goplot package in R for data analyses^43,44^.

### Protein-protein interaction (PPI) analysis

Information on protein experimental interactions and prediction was obtained using the STRING database (version 10.5)^45^. Coexpression experiments, gene fusion, neighborhoods, text mining and co-occurrence as the prediction methods were performed for these databases. Except for these, we used combinatorial scores to show the interaction of protein pairs. In the current study, a combined score >0.9 was set as the threshold^46,47^ to pick up the hub genes in the network after DEGs were mapped to PPIs. We used degrees to show the role of protein nodes in the network. Network modules may have a specific biological significance, so it is usually the core of protein network. We used Cytoscape software package (version 3.61) to detect main clustering modules. The most notable clustering modules, were identified using the Molecular Complex Detection (MCODE) app^48,49^. Subsequently, to identify the KEGG pathway for DEGs enrichment, we also employed the clusterProfiler package in R and set the threshold at EASE to ≤0.05, count ≥ 2. The MCODE score was set at the cutoff value of >6.

### Network construction of competing endogenous RNAs (ceRNAs)

Due to the potential interaction among mRNAs, miRNAs and circRNAs, we constructed a ceRNA network for further analysis. We used the starBase (V3.0) and circinteractome databases^50,51^ to predict interactions between circRNA and miRNA. The relationship between microRNAs and RNAs was predicted by miRwalk and DIANA TOOLS^52,53^. Since the results of starBase and DIANA TOOLs databases are derived from CLIP-Seq (HITS-CLIP, PAR-CLIP, iCLIP, CLASH), their predictions of the ceRNA network interactions was highly reliable. The retrieval terms are as follows: (“hsa-miR-296” or “miR-296” or “has-miR-296-3p” or “hsa-miR-21” or “miR-21” or “hsa-miR-21-3p” or “hsa-miR-21-5p” or “miR-21-3p” or “miR-21-5p” or “hsa-miR-361” or “miR-361” or “hsa-miR-361-5p” or “hsa-miR-375” or “miR-375”) and (“noncoding RNA” or “ncRNA” or “noncoding RNA” or “non-coding RNA” or “circ RNA” or “circular RNA”). If there was no interaction between DEGs and DEMis, it was removed after GO, DO, KEGG PPI and MCODE analyses. Similar methods were used to predict the interaction between circRNAs and miRNAs. Finally, circRNAs were found intersected of the four DEMis, and the ceRNA network was visualized by Cytoscape app.

### Patients

All patients in this study were hospitalized in the First Affiliated Hospital of Guangxi Medical University from Jan. 1, 2015 to Dec. 31, 2017. Patients were diagnosed with CAD, ischemic stroke, type 2 diabetes, dyslipidemia, essential hypertension and obesity. When choosing patients, we should first ensure that they met the main diagnosis and tried to control for patients with other illnesses. Group 1 contained 2158 subjects who suffered from arteriosclerosis-related diseases. Group 2 included hospitalized patients with chest complaints.

The normal values were defined as serum total cholesterol (TC) levels of 3.10–5.17 mmol/L, serum triglyceride (TG) levels of 0.56–1.70 mmol/L, serum low-density lipoprotein cholesterol (LDL-C) levels of 2.70–3.20 mmol/L, serum high-density lipoprotein cholesterol (HDL-C) levels of 0.91–1.81 mmol/L, serum apolipoprotein (Apo) A1 levels of 1.00–1.78 g/L, serum ApoA1 levels of 0.63–1.14 g/L, and ApoA1/B levels of 1.00–2.50^54^. Type 2 diabetes mellitus was diagnosed according to the World Health Organization (WHO) diagnostic criteria^55^. According to WHO criteria, if serum TG levels were ≥1.7 mmol/L and HDL-C levels <0.9 mmol/L for men or <1.0 mmol/L for women, the patient was diagnosed with dyslipidemia^56^. The 2017 ACC/AHA guidelines were used to define the management of hypertension^57^. According to the calculation of BMI, normal weight was defined as <24 kg/m^2^, overweight was defined as 24–28 and obesity was defined as >28 kg/m^2^ ^58^. All patients with CAD needed to be diagnosed using coronary angiography, which was examined by two experienced experts. CAD are defined when stenosis of at least one of the three main coronary arteries and their branches (branched diameter >2 mm) exceed 50%^59^. After rigorous examination, the TOAST (Trial of Org 10172 in Acute Stroke Treatment) criteria were used to measure compliance with ischemic stroke (IS) standards, including magnetic resonance imaging (MRI), computed tomography and neurological tests^60^. Two subtypes of the TOAST criteria, small-vessel occlusion and/or large-artery atherosclerosis, were included for IS patients.

Standard medical questionnaires were used to obtain medical history and general information of the subjects. CAD was determined by the results of clinical physical examination, laboratory test and coronary angiography. Exclusion criteria from this study included previously diagnosed patients with CAD, type 1 diabetes, congenital heart disease and major organ dysfunction. We informed the subjects of the experimental steps of this study and obtained their consent and signature confirmation. The design of the study was in line with the Helsinki Declaration (http://www.wma.net/en/30 publications/10 politics/b3/) and was agreed on after discussion by the Ethics Committee of the First Affiliated Hospital of Guangxi Medical University (No: Lunshen-201 l-KY-Guoji-001; March 7, 2011). The results of laboratory biochemical examination were obtained during hospitalization, and clinical data collection and laboratory biochemical examination were carried out according to prior study^61^.

### RT-quantitative PCR (qPCR)

Blood samples were taken from patients who fasted, every other morning. Samples were placed in an EDTA coated tube at 5 ml in volume and centrifuged at room temperature for 15 minutes, 3000 g/min. The above steps are accomplished by centrifuging at 1200 g/min for 15 minutes to remove the fragmented cells in the environment of 4 C. We used TRIzol (Invitrogen, Carlsbad, CA, USA) reagent to isolate total RNA from blood samples according to manufacturer’s instructions. Next, samples were further treated with RNase R (Epic Center, Inc., Madison, WI, USA). A Nano Drop ND-1000 spectrophotometer (Nano Drop Thermo, Wilmington, DE) was used to determine extracted RNA quantity and quality. A reverse transcriptase kit (TIANGEN; catalog number: KR211, China) was used to generate cDNA according to manufacturer’s instructions. The expression levels of the circRNAs, miRNAs and mRNAs were evaluated by qPCR using SYBR Green assays. Primers (Supplementary Table 4) were commercially designed to amplify transcription products. A mixed liquid of 10 µ1 including 5 μl 2 × Master Mix, 0.5 μl of Reverse Primer (10 μM), 2 μl of cDNA, 0.5 μl of Forward Primer (10 μM) and 2 μl of double distilled water was obtained and PCR was performed under this reaction system. The reaction was incubated at 95 °C for 15 minutes, at 94 °C for 25 seconds, at 60 °C for 30 seconds, and at 72 °C for 34 seconds. All reactions were performed in duplicate. Ct values were averaged for each sample after two independent PCR experiments. GAPDH served as a housekeeping gene for circRNA and mRNA whereas U6 served as a housekeeping gene for miRNAs^62,63^. Relative quantification using the 2^−∆∆CT^ formula was used for data analyses.

### Cell culture

Human monocytic THP-1 cells and HASMCs were purchased from ATCC (Manassas, VA). THP-1 cells were grown in RPMI 1640 medium (R8758, Sigma-Aldrich, St. Louis, MO, USA) supplemented with 1% penicillin/streptomycin solution (Gibco, Carlsbad, CA) and 10% fetal bovine serum (FBS, Gibco, Carlsbad, CA). To perform experiments, cells were seeded into 12-well culture plates and incubated with 100 ng/ml phorbol 12-myristate 13-acetate (PMA, Sigma, USA). After 48 h, the morphology of differentiated macrophages was observed. HASMCs cells were cultured in Dulbecco’s modified Eagle’s medium (DMEM) supplemented with 0.5 ng/ml human epidermal growth factor (EGF), 2 ng/ml human fibroblast growth factor-2 (FGF-2), 10% fetal bovine serum (FBS), 1% penicillin-streptomycin and 5 g/ml insulin. All cells were incubated at 37 °C with 5% CO_2_ in a humidified chamber containing.

The pHB-circBasic™ circular RNA cloning kit (Hanbio, Shanghai, China) was used to generate the Circ-YOD1 overexpression vector. The front cir-signal and back cir-signal had been designed, and the downstream region of the CMV promoter had also been added to the pHB-circBasic™ vector. In brief, the cDNA encoding the liner form of the *YOD1* transcript in these two cells was amplified using primers 5′-CAATGGGGTGTATGGGTGGG-3′ and 5′-CCGGGTGACGAGAAAATGCT-3′, and then, the PCR product was purified by using a Gel Extraction Kit (Omega Bio-tek, Doraville, GA, USA). First, we used circular primers to amplify the purified PCR product. Then, the fragments were purified twice for PCR and inserted into the site between the front cir-signal frame and back cir-signal frame in the pHB-circBasic™ vector. Finally, THP-1 cells and HASMCs were transfected with circ-YOD1 plasmids using Turbojet transfection reagent (Thermo, Waltham, MA, USA). siRNAs were transfected into cells using a riboFECT ™ CP Transfection Kit (RiboBio, Guangzhou, China).

### Statistical analysis

SPSS 25.0 statistical software package (SPSS Inc. Chicago, IL, USA) was used for statistical analyses, which was validated by Prism 8.0 (GraphPad Software). A Chi-square test was used to compare in ratios between the two groups. Continuous data were shown as the mean ± SD^64^ for normally distributed data. Due to the skewed distribution of TG, median and quartile spacing were used to compare the differences. Mann-Whitney nonparametric tests and analysis of covariance (ANCOVA) were performed for comparing continuous data^65^. ANCOVA is an ideal statistical method for studying the interaction between genes and the environment. It is characterized by the ability to correct for confounding factors^66,67^. R software (version 3.5.0) was used for further bioinformatic analyses. The receiver operating characteristic (ROC) curve was used to evaluate the diagnostic sensitivity of plasma miRNAs and circRNAs in patients with CAD. The areas under the curves (AUC) were calculated and compared. All tests were conducted as two-sided and statistical significance was defined as *P* < 0.05.

## Supplementary information

Supplementary Figure

Supplementary Table 3

Supplementary Table 4

Supplementary Table 1

Supplementary Table 2
