# Supplementary material for: A novel circRNA-miRNA-mRNA network identifies circ-YOD1 as a biomarker for coronary artery disease
Source: Sci Rep. 2019 Dec 4;9:18314. doi: 10.1038/s41598-019-54603-2 (PMC6892882; doi:10.1038/s41598-019-54603-2)
Supplement: Supplementary file 1 — Supplementary Figure [file 41598_2019_54603_MOESM1_ESM.pdf]

A novel circRNA-miRNA-mRNA network identifies circ-YOD1 as a biomarker for coronary artery disease

Liu Miao[1] , Rui-Xing Yin ID[1,2,3]* , Qing-Hui Zhang[1] , Pei-Juan Liao[1] , Yong Wang[1] , Rong-Jun Nie[1] , Hui Li[4]

1. Department of Cardiology, Institute of Cardiovascular Diseases, The First Affiliated Hospital, Guangxi Medical University, 6 Shuangyong Road, Nanning 530021, Guangxi, China.
2. Guangxi Key Laboratory Base of Precision Medicine in Cardio-cerebrovascular Disease Control and Prevention, 6 Shuangyong Road, Nanning 530021, Guangxi, China.
3. Guangxi Clinical Research Center for Cardio-cerebrovascular Diseases, 6 Shuangyong Road, Nanning 530021, Guangxi, China.
4. Clinical Laboratory of the Affiliated Cancer Hospital, Guangxi Medical University, 71 Hedi Road, Nanning 530021, Guangxi, China.

* Corresponding author: Rui-Xing Yin; yinruixing@163.com

Department of Cardiology, Institute of Cardiovascular Diseases, The First Affiliated Hospital, Guangxi Medical University, Nanning 530021, Guangxi, PR China

ID: 0000-0001-7883-4310

Running title: circ-YOD1 is a biomarker for coronary artery disease

dr.miaoliu@qq.com

yinruixing@163.com

zhangqinghuixx@163.com

peijuanliao@163.com

wangyong007-007@163.com

nrj2001@163.com

nnlihui@163.com

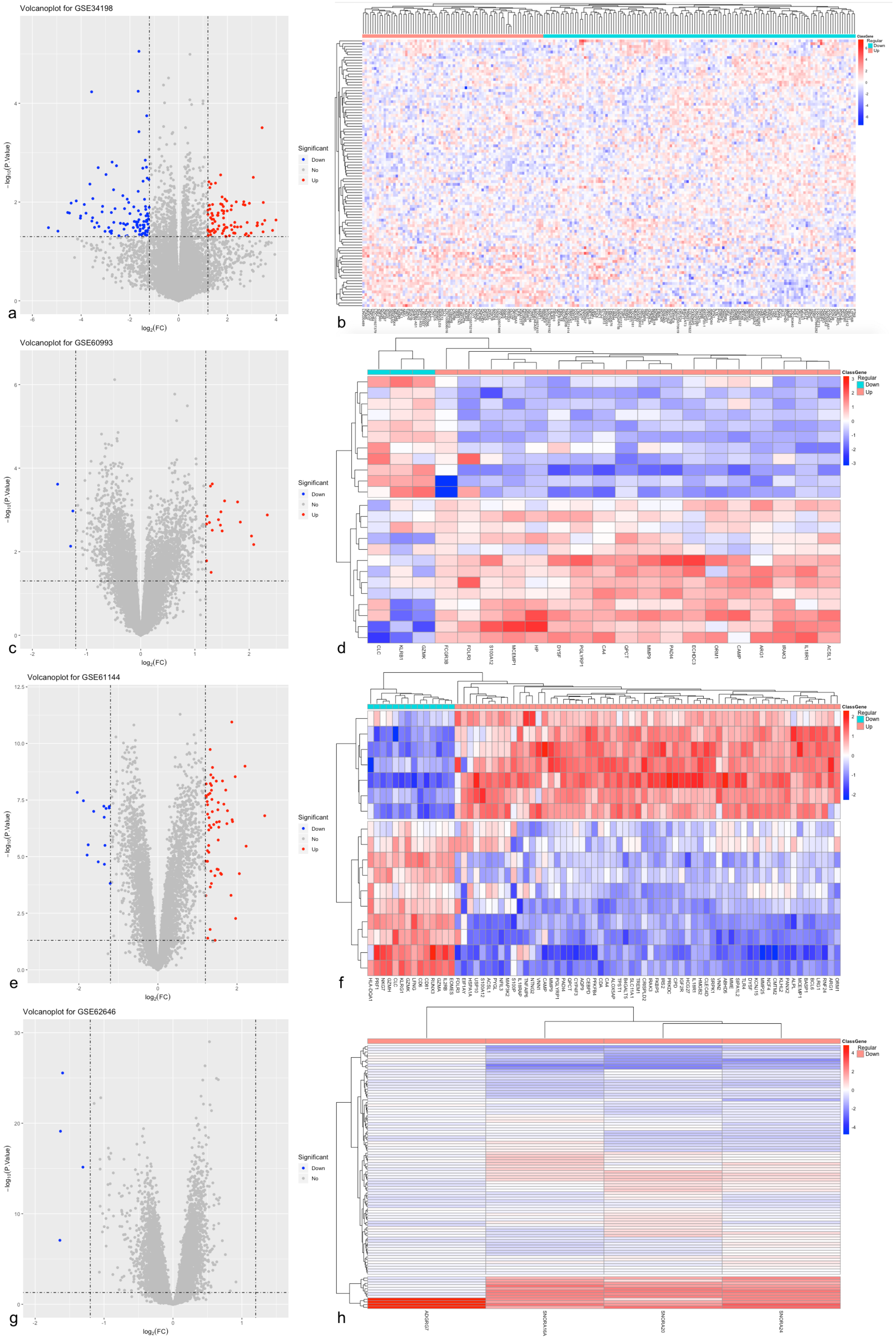

Supplementary Figure 1. The heatmap and volcano plot for DEGs. The two vertical lines are the 1.2-fold change boundaries, and the horizontal line is the statistical

significance boundary ($p < 0.05$). Items with statistical significance and upregulation are marked with red dots, and downregulation is marked with green dots in a volcano plot (a, c, e, g). For the heatmap (b, d, f, h), the red strip represents high relative expression, and the blue strip represents low relative expression. Upregulated genes are in the red cluster, and downregulated genes are in the green cluster.

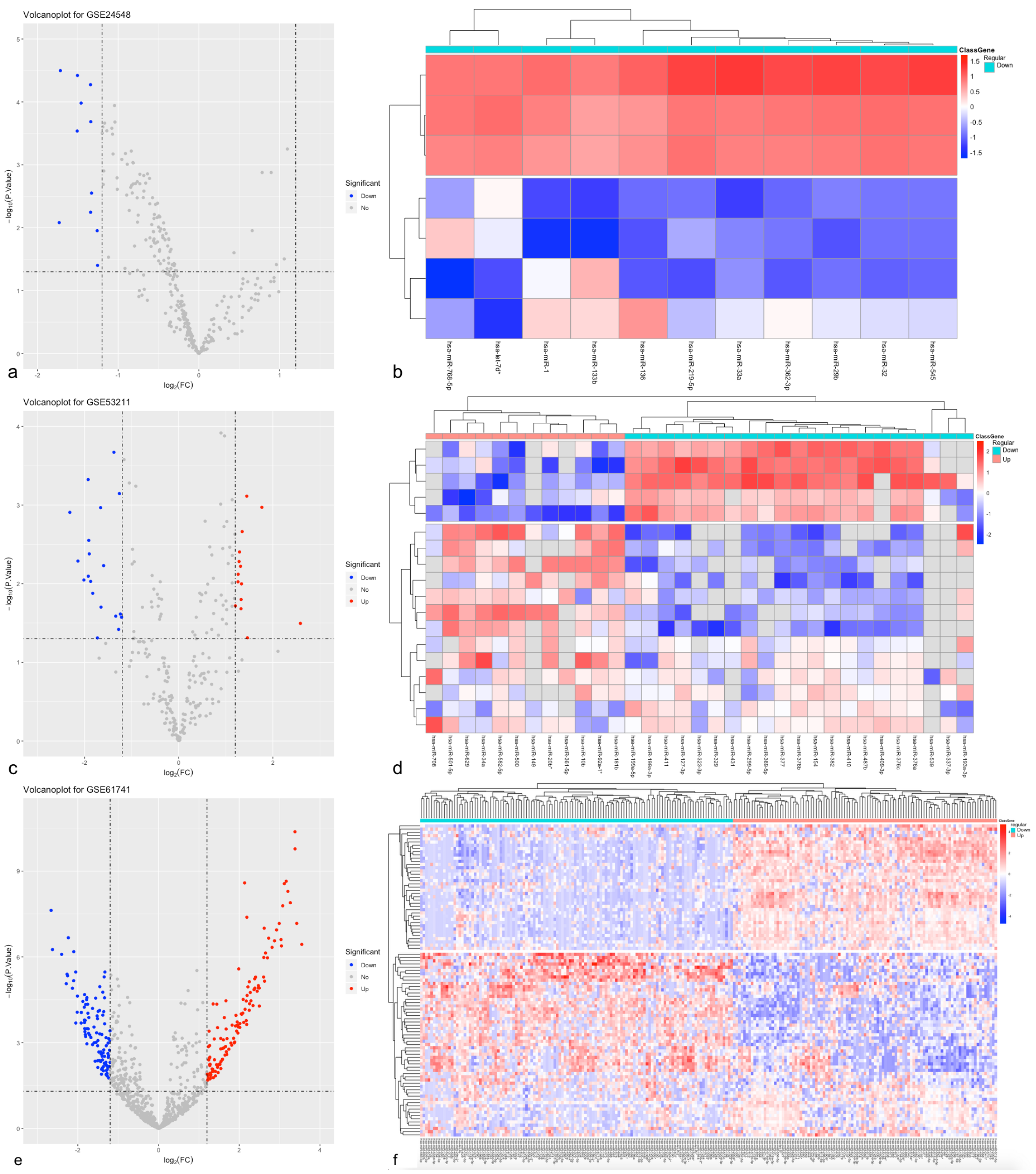

Supplementary Figure 2. The heatmap and volcano plot for DEMis. The two vertical

lines are the 1.2-fold change boundaries, and the horizontal line is the statistical significance boundary ($p < 0.05$). Items with statistical significance and upregulation are marked with red dots, and downregulation is marked with green dots in a volcano plot (a, c, e). For the heatmap (b, d, f), the red strip represents high relative expression, and the blue strip represents low relative expression. Upregulated genes are in the red cluster, and downregulated genes are in the green cluster.
