# Supplementary material for: A novel circRNA-miRNA-mRNA network identifies circ-YOD1 as a biomarker for coronary artery disease
Source: Sci Rep. 2019 Dec 4;9:18314. doi: 10.1038/s41598-019-54603-2 (PMC6892882; doi:10.1038/s41598-019-54603-2)
Supplement: Supplementary file 2 — Supplementary Table 3 [file 41598_2019_54603_MOESM2_ESM.pdf]

A novel circRNA-miRNA-mRNA network identifies circ-YOD1 as a biomarker for coronary artery disease

Liu Miao[1] , Rui-Xing Yin ID[1,2,3]* , Qing-Hui Zhang[1] , Pei-Juan Liao[1] , Yong Wang[1] , Rong-Jun Nie[1] , Hui Li[4]

1. Department of Cardiology, Institute of Cardiovascular Diseases, The First Affiliated Hospital, Guangxi Medical University, 6 Shuangyong Road, Nanning 530021, Guangxi, China.
2. Guangxi Key Laboratory Base of Precision Medicine in Cardio-cerebrovascular Disease Control and Prevention, 6 Shuangyong Road, Nanning 530021, Guangxi, China.
3. Guangxi Clinical Research Center for Cardio-cerebrovascular Diseases, 6 Shuangyong Road, Nanning 530021, Guangxi, China.
4. Clinical Laboratory of the Affiliated Cancer Hospital, Guangxi Medical University, 71 Hedi Road, Nanning 530021, Guangxi, China.

* Corresponding author: Rui-Xing Yin; yinruixing@163.com
Department of Cardiology, Institute of Cardiovascular Diseases, The First Affiliated Hospital, Guangxi Medical University, Nanning 530021, Guangxi, PR China
ID: 0000-0001-7883-4310

Running title: circ-YOD1 is a biomarker for coronary artery disease

dr.miaoliu@qq.com
yinruixing@163.com
zhangqinghuixx@163.com
peijuanliao@163.com
wangyong007-007@163.com
nrj2001@163.com
nnlihui@163.com

Supplementary Table 3. Basic information of the microarray datasets from GEO

Data source	Platform	Country	Contact name	Type	Ctrl (n)	Case (n)	Ref.
GSE34198	GPL6102	Czech Republic	Michal Kolář	mRNA	48	49	[14]
GSE62646	GPL6244	Poland	Beata Burzynska	mRNA	14	84	[15]
GSE60993	GPL6884	South Korea	JUNGWOO EUN	mRNA	7	17	[16]
GSE61144	GPL6106	South Korea	JUNGWOO EUN	mRNA	10	7	[16]
GSE24548	GPL8227	Italy	Gerolamo Lanfranchi	miRNA	3	4	No
GSE53211	GPL18049	Germany	Julia Osipova	miRNA	4	9	No
GSE61741	GPL9040	Germany	Andreas Keller	miRNA	34	62	[17]

n, numbers. Ctrl, Control. Ref, Reference. The experimental designed as case-control study and the case disease was related to CAD. All samples analyzed by the microarray were peripheral blood.
