# Supplementary material for: A novel circRNA-miRNA-mRNA network identifies circ-YOD1 as a biomarker for coronary artery disease
Source: Sci Rep. 2019 Dec 4;9:18314. doi: 10.1038/s41598-019-54603-2 (PMC6892882; doi:10.1038/s41598-019-54603-2)
Supplement: Supplementary file 3 — Supplementary Table 4 [file 41598_2019_54603_MOESM3_ESM.pdf]

A novel circRNA-miRNA-mRNA network identifies circ-YOD1 as a biomarker for coronary artery disease

Liu Miao[1], Rui-Xing Yin ID[1,2,3]*, Qing-Hui Zhang[1], Pei-Juan Liao[1], Yong Wang[1], Rong-Jun Nie[1], Hui Li[4]

1. Department of Cardiology, Institute of Cardiovascular Diseases, The First Affiliated Hospital, Guangxi Medical University, 6 Shuangyong Road, Nanning 530021, Guangxi, China.
2. Guangxi Key Laboratory Base of Precision Medicine in Cardio-cerebrovascular Disease Control and Prevention, 6 Shuangyong Road, Nanning 530021, Guangxi, China.
3. Guangxi Clinical Research Center for Cardio-cerebrovascular Diseases, 6 Shuangyong Road, Nanning 530021, Guangxi, China.
4. Clinical Laboratory of the Affiliated Cancer Hospital, Guangxi Medical University, 71 Hedi Road, Nanning 530021, Guangxi, China.

* Corresponding author: Rui-Xing Yin; yinruixing@163.com

Department of Cardiology, Institute of Cardiovascular Diseases, The First Affiliated Hospital, Guangxi Medical University, Nanning 530021, Guangxi, PR China

ID: 0000-0001-7883-4310

Running title: circ-YOD1 is a biomarker for coronary artery disease

dr.miaoliu@qq.com

yinruixing@163.com

zhangqinghuixx@163.com

peijuanliao@163.com

wangyong007-007@163.com

nrj2001@163.com

nnlihui@163.com

Supplementary Table 4: The primer for RT-PCR

Name	F	R
U6	CTCGCTTCGGCAGCACA	AACGCTTCACGAATTTGCGT
GAPDH	AGGTCGGTGTGAACGGATTTG	GGGGTCGTTGATGGCAACA
has-miR-21-3p	ACAGCCCAUCGACUGGUGUUG	CCAGTCTCAGGGTCCGAGGTATTC
has-miR-296-3p	GGAGAGCCUCCACCCAACCCUC	CCAGTCTCAGGGTCCGAGGTATTC
has-miR-361-5p	GUACCCCUGGAGAUUCUGAUAA	CCAGTCTCAGGGTCCGAGGTATTC
has-miR-375	GGUUUGUGAGGGGCUCGUCGC	CCAGTCTCAGGGTCCGAGGTATTC
BCL6	GAGCCACAGGTTGCAAATCC	CCGCGTTCCTTATGTGCAAG
FBXL1	CCTGGGAATGAGGGTTGGTC	CCCCAACCATCGGAAGTGAA
FCGR3B	CCCATCCCTTTGTGGGAGTC	CCTGTTCTATGGTGGGGCTC
MMP9	TTTAGCAAACGTAGGGGCGG	CTTCACGTCGAACCTGCGG
Circ-YOD1	AAGGTCGCCATTTTGGAGTC	CAAAACATGGGTGCCGTCCT
